# Analysis of copy number loss of the ErbB4 receptor tyrosine kinase in glioblastoma

**DOI:** 10.1371/journal.pone.0190664

**Published:** 2018-01-17

**Authors:** DeAnalisa C. Jones, Adriana Scanteianu, Matthew DiStefano, Mehdi Bouhaddou, Marc R. Birtwistle

**Affiliations:** Department of Pharmacological Sciences, Icahn School of Medicine at Mount Sinai, New York, New York, United States of America; Seoul National University College of Pharmacy, REPUBLIC OF KOREA

## Abstract

Current treatments for glioblastoma multiforme (GBM)—an aggressive form of brain cancer—are minimally effective and yield a median survival of 14.6 months and a two-year survival rate of 30%. Given the severity of GBM and the limitations of its treatment, there is a need for the discovery of novel drug targets for GBM and more personalized treatment approaches based on the characteristics of an individual’s tumor. Most receptor tyrosine kinases—such as EGFR—act as oncogenes, but publicly available data from the Cancer Cell Line Encyclopedia (CCLE) indicates copy number loss in the ERBB4 RTK gene across dozens of GBM cell lines, suggesting a potential tumor suppressor role. This loss is mutually exclusive with loss of its cognate ligand NRG1 in CCLE as well, more strongly suggesting a functional role. The availability of higher resolution copy number data from clinical GBM patients in The Cancer Genome Atlas (TCGA) revealed that a region in Intron 1 of the ERBB4 gene was deleted in 69.1% of tumor samples harboring ERBB4 copy number loss; however, it was also found to be deleted in the matched normal tissue samples from these GBM patients (n = 81). Using the DECIPHER Genome Browser, we also discovered that this mutation occurs at approximately the same frequency in the general population as it does in the disease population. We conclude from these results that this loss in Intron 1 of the ERBB4 gene is neither a *de novo* driver mutation nor a predisposing factor to GBM, despite the indications from CCLE. A biological role of this significantly occurring genetic alteration is still unknown. While this is a negative result, the broader conclusion is that while copy number data from large cell line-based data repositories may yield compelling hypotheses, careful follow up with higher resolution copy number assays, patient data, and general population analyses are essential to codify initial hypotheses prior to investing experimental resources.

## Introduction

The ERBB/HER family of receptor tyrosine kinases (RTK’s) includes EGFR/ERBB1/HER1, ERBB2/HER2, ERBB3/HER3, and ERBB4/HER4 [1–5]. Their activation by ligand binding followed by homo- and hetero-dimerization leads to activation of multiple mitogenic and survival pathways, such as the MAPK signaling pathway, which can drive cell proliferation and cell survival [1–3,5]. It is known that amplification in the gene copy number of ERBB/HER genes leads to overexpression and the sustained cell proliferation and survival in many cancers [1,2,4,5]. Overexpression of EGFR has been observed in many primary tumor types including lung, pancreas, breast, and glioblastoma while overexpression of HER2 has primarily been observed in breast and ovarian cancers [1,2,4]. Mutations such as these can be exploited when developing targeted cancer therapies [1–3,5]. For example, because EGFR is known to be overexpressed in non-small cell lung cancer (NSCLC), the most common type of lung cancer [5,6], and NSCLC is treated using gefitinib—a kinase inhibitor that binds to the intracellular tyrosine kinase domain of EGFR and inhibits the signaling that drives cell proliferation and survival [6]. Amplification in HER2 copy number is observed in 20–30% of breast carcinomas [1]. Patients with this copy number variation are treated with trastuzumab—a monoclonal antibody that binds to the extracellular domain of HER2 to inhibit signaling that drives cell proliferation and survival [1,6].

While much information is available about EGFR and HER2 signaling in cancer, less is known about the role of ERBB4. ERBB4 binds several ligands including betacellulin, HB-EGF, and epiregulin that also bind to EGFR [2] but additionally other ligands such as neuregulin 1–4 (NRG1, NRG2, NRG3, and NRG4) [7]. ERBB4 is essential to cardiac, mammary, and neural development [2,8] and is implicated in schizophrenia [9]. With regards to signaling and cancer, ERBB4 activates several of the same downstream proteins as EGFR—such as CBL, STAT5, and SHC [2] but also strongly activates PI3K signaling [2,3]. Its overexpression has been associated with melanoma, medulloblastoma, and breast cancer progression [4,8]. However, it remains unclear what role, if any, ERBB4 plays in the progression of gliomas.

Copy number variations (CNV’s) are generally accepted to be any genomic variations greater than 50 bp in length that alter the amount of DNA content of a gene [10,11]. CNV’s are commonly termed deletions when DNA content of a gene is lost and amplifications when DNA content is gained. CNV’s can play an important role in human disease—by altering the structure or abundance of transcripts and proteins, for example—or can have no phenotypic effect [10,11]. Examples in many human cancers include copy number loss of the gene that codes for the tumor suppressor protein PTEN [4,12] and copy number gain in the gene that codes for the proto-oncogene EGFR [2]. Loss of a tumor suppressor gene, such as PTEN, and amplification of a proto-oncogene, such as EGFR, both lead to cancer progression [2,4,12]. When a tumor suppressor is lost, it is no longer able to quell cell proliferation or induce cell death [12]. When a proto-oncogene is gained, amplified cell proliferation or inability to induce death occurs [1,2]. While a large volume of publicly available copy number data is generated using microarray and next-generation sequencing technologies [13,14,15], much work remains in processing this data and interpreting the functional impacts of specific CNV’s in human disease [10].

Here, we use publicly available data to explore copy number variation of ERBB4 in gliomas. Data from the Cancer Cell Line Encyclopedia (CCLE) suggests copy number loss of ERBB4 may be significant in glioma. However, subsequent follow up in The Cancer Genome Atlas (TCGA) and the DECIPHER Genome Browser demonstrate that the CCLE indications seem to be artifacts, which may be due to a combination of cell line models and low resolution copy number variation measurements. This study cautions on the importance of following up on findings from copy number data in cell lines—particularly those data generated using an aCGH microarray data—and outlines a comprehensive approach to using publicly available copy number data to gain insight into the potential functional impact of CNV’s in cancer.

## Methods

### Data curation

DNA Copy Number (41.6GB) Affy SNP data in the form of copy number by gene for 60 glioma cell lines was downloaded from the Cancer Cell Line Encyclopedia (CCLE) data portal at https://portals.broadinstitute.org/. Copy Number Segment data from the Affymetrix SNP 6.0 platform for 526 GBM tumor and matched normal tissue samples was downloaded from The Cancer Genome Atlas (TCGA) data portal at https://portal.gdc.cancer.gov. All downloaded data from CCLE and TCGA can be found in S1 Table. The DECIPHER Genome Browser compiles data on the general, healthy population from various sources into one online tool (Firth, H. V. et al. PMC, 2009). Raw copy number data of healthy individuals from studies included in DECIPHER was not downloaded or analyzed by our group as was done with the CCLE and TCGA data. The frequency of copy number variations across datasets is already summarized for us in the genome browser. We only had to query our CNV of interest using its chromosomal location (Ch. 2:213186816–213191560) for the Population: Copy-Number Variants Affy6 consolidated data set of the browser, as outlined in the methods section of the manuscript. This query was performed on April 20, 2017. Copy number data from all three databases was generated using the Affymetrix SNP 6.0 microarray system.

### Data processing

Raw microarray data describes each segment of the genome by a chromosome number and base pair range and a segment mean is assigned to each. This raw data can be converted to copy number by gene by locating the segment of the genome that contains the gene and then using the following formula to convert the segment mean (SM) to copy number (CN) [16]:

$$SM={log}_{2}(\frac{CN}{2})$$

Downloaded data from CCLE was already converted into copy number by gene. Downloaded data from TCGA needed to be converted to copy number by gene, which was done using the above equation. All gene locations within the genome were taken from the UCSC Genome Browser on Human Feb. 2009 (GRCh37/hg19). Based on the normalized segment mean distribution, copy number loss was defined as segment mean < -0.6, which is equivalent to a copy number of approximately 1.3195. Copy number gain was defined as segment mean > 0.6, or copy number of approximately 3.0314.

## Results

### Gene copy number distribution in CCLE

A long-term goal in our lab is to construct mechanistic mathematical models of glioma cell signaling that integrate commonly mutated signaling pathways and are tailored to an individual tumor’s genomic, transcriptomic, and proteomic data [14,17–23]. Public databases are a potentially valuable source of data for this research direction [14].

**Fig 1:**
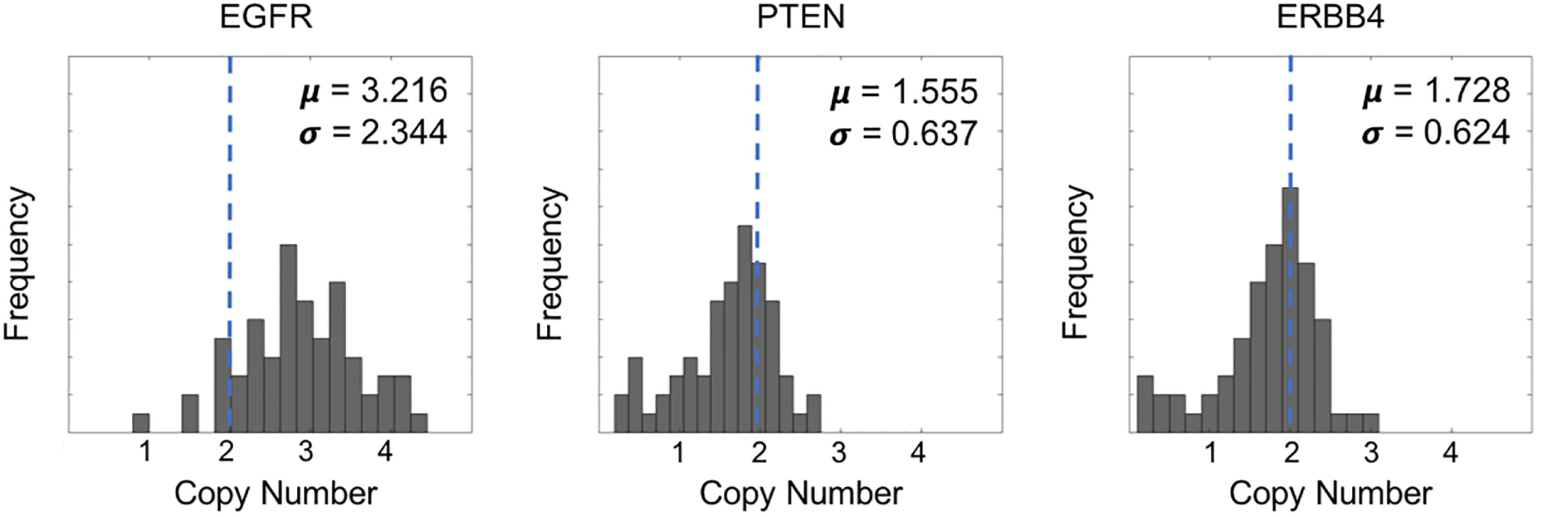
Gene copy number distribution in CCLE. Gene copy number distributions of the ERBB4, EGFR, and PTEN genes across all glioma cell lines in CCLE (n = 60). The ERBB4 gene copy number distribution is shifted to the left of normal copy number of 2 for diploid cells, signifying copy number loss or deletion. Copy number data for EGFR and PTEN was analyzed to confirm what distribution properties are expected of a known oncogene (EGFR) and a known tumor supressor (PTEN). An oncogene, which is amplified in cancer, exhibits a right-shifted distribution while a tumor suppressor, which is lost in cancer, exhibits a left-shifted distribution.

With this goal in mind, an initial analysis of copy number data from 60 glioma cell lines from the CCLE database revealed that the copy number distribution for ERBB4 is shifted to the left of normal copy number of 2 for diploid cells, signifying copy number loss or deletion (Fig 1). EGFR and PTEN were used as positive and negative controls respectively, and ERBB4 copy number is similar to the known tumor suppressor PTEN. This result is counter to what might be expected from a member of the ERBB family—as we can see from the right-shifted EGFR copy number distribution—and suggested that ERBB4 may be acting as a tumor suppressor in gliomas.

### Mutually exclusive copy number loss of ERBB4 and NRG1 genes

**Fig 2:**
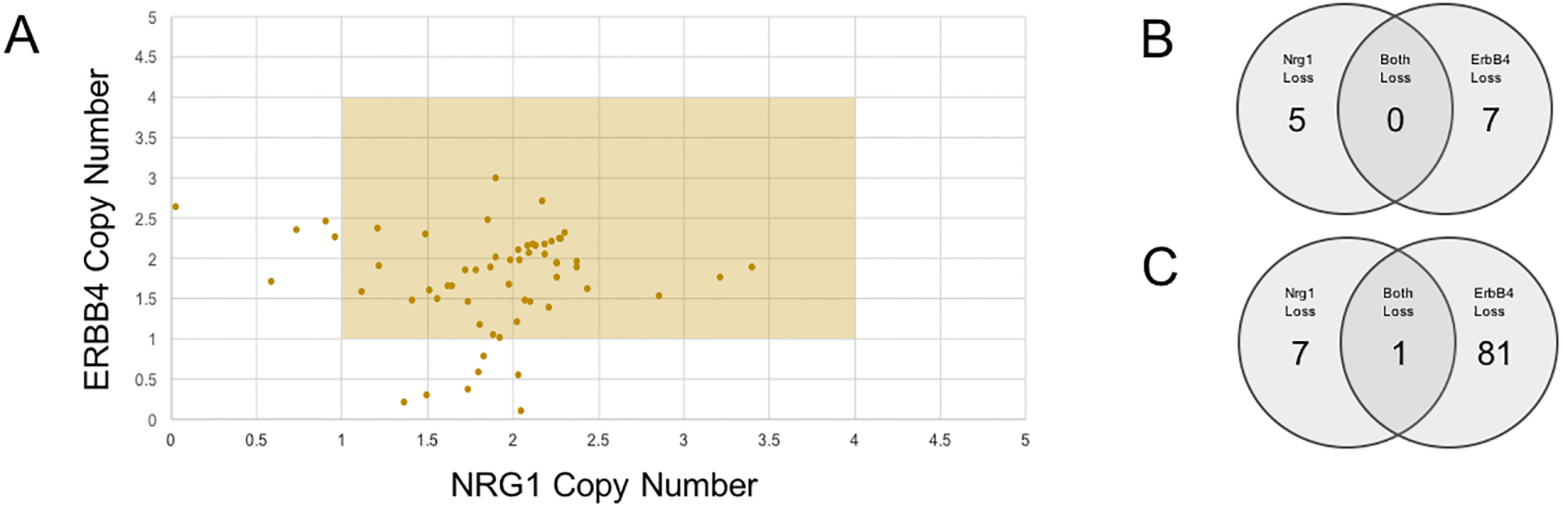
Mutually exclusive copy number loss of ERBB4 and NRG1 genes. **(a)** Shown is ERBB4 copy number versus NRG1 copy number for each cell line where the highlighted region represents diploidy. Across all glioma cell lines in CCLE, ERBB4 and NRG1 copy number loss never occur simultaneously. **(b)** Across all glioma cell lines in CCLE (n = 60), ERBB4 copy number loss occurs in 7 cell lines and NRG1 occurs in 5 cell lines. Again, loss of both genes never occurs in the same cell line. **(c)** Across all GBM tumor samples from TCGA (n = 526), ERBB4 copy number loss occurs in 81 patients and NRG1 occurs in 7 patients. Both genes are only lost in 1 patient.

If ERBB4 is behaving as a tumor suppressor, only the receptor or the ligand—but not both—needs to be missing in order for there to be loss of tumor suppressor activity i.e. cancer progression. Therefore, we asked whether there was mutual exclusivity in copy number loss between ERBB4 and its endogenous ligand in the central nervous system, neuregulin-1 (NRG1). Copy number analysis of all glioma cell lines in CCLE revealed that there is a potential mutually exclusive relationship between loss in ERBB4 and loss in NRG1 as ERBB4 and NRG1 copy number loss never occur simultaneously (Fig 2a and 2b).

### ERBB4 copy number loss in TCGA

Our preliminary analyses in glioma cell lines were expanded to GBM tumor samples from patients in TCGA to address whether or not the results from cell lines translate to clinical GBM patients. A nearly mutually exclusive relationship (albeit not as strong as in CCLE) was observed between ERBB4 and NRG1 copy number loss; however, loss of ERBB4 is strongly favored compared to NRG1 loss, as opposed to more parity in CCLE. While NRG1 loss was observed in only 7 of the tumor samples from 526 GBM patients in TCGA, ERBB4 loss was observed in 81 samples (Fig 2c). NRG1 is the primary ligand for ERBB4 in the CNS; however, ERBB4 can also bind NRG2, NRG3, and betacellulin (BTC). Upon looking into the copy number loss of NRG2, NRG3, and BTC in glioma cell lines from CCLE, we found that no copy number loss was observed across glioma cell lines for NRG2 and BTC and copy number loss of NRG3 occurred in approximately 12% of glioma cell lines. An additional analysis of ERBB4 loss in glioma cell lines was later explored in the HGCC gliomasphere database [24]. This analysis showed normal copy number for ERBB4 across 48 GBM cell lines (S1 Fig).

**Fig 3:**
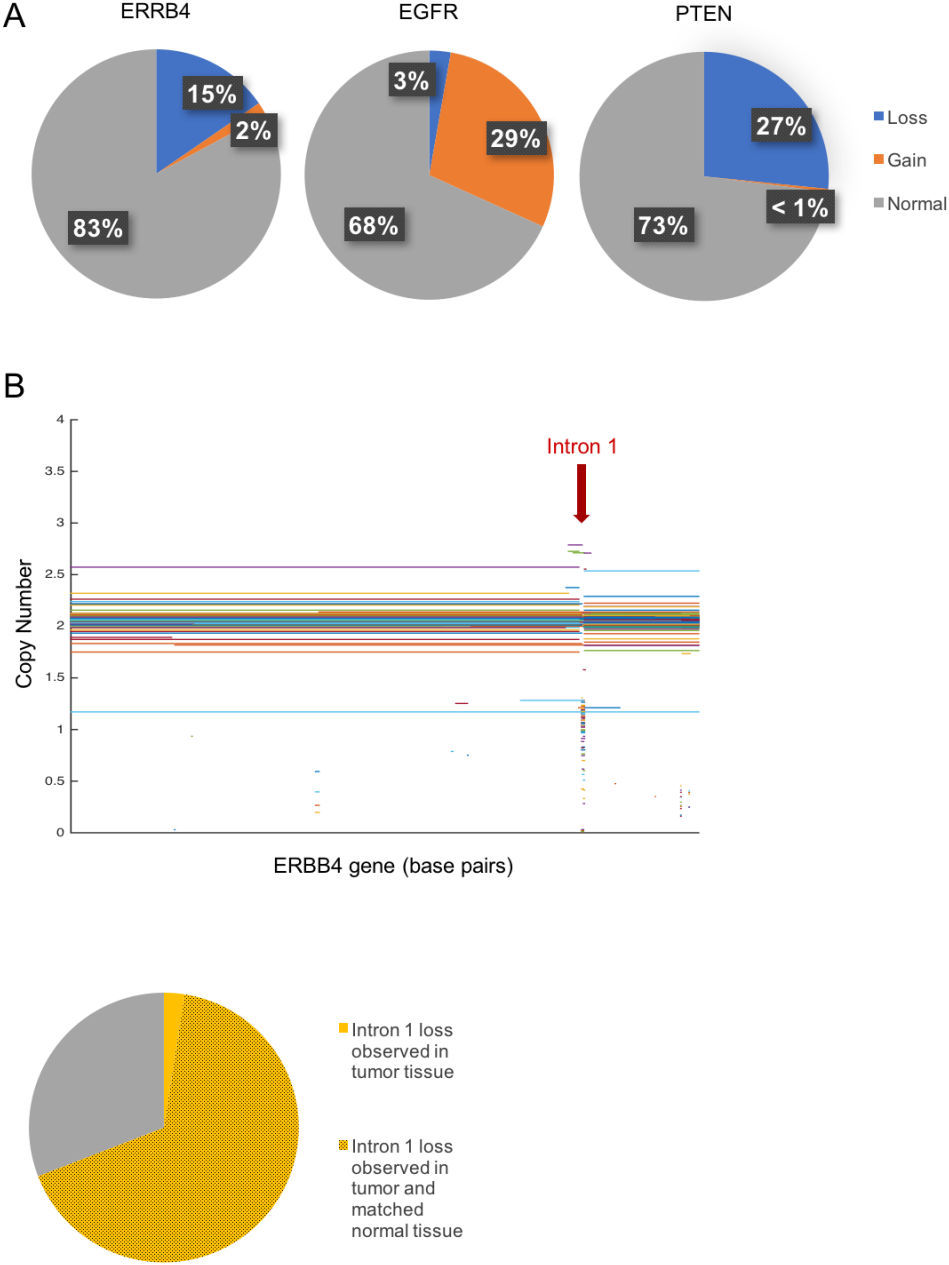
Frequency of copy number loss in TCGA. **(a)** Shown are the percentages of normal copy number, copy number loss, and copy number gain observed for ERBB4, EGFR (a known oncogene in GBM), and PTEN (a known tumor suppressor in GBM) in GBM tumor samples from TCGA (n = 526). The ERBB4 gene was lost in 15.4% of samples. When compared to EGFR and PTEN, this frequency of loss behaves similarly to that of known GBM tumor suppressor PTEN. **(b)** Here, copy number values from segmented copy number data are mapped to segments of nucleotides within the ERBB4 gene. Copy number loss in ERBB4 appears to be localized to one region located in Intron 1 of the gene. **(c)** Of the tumor samples where ERBB4 loss was observed (n = 81), 69.1% of the loss was observed in a localized region within intron 1 of the ERBB4 gene. Of the tumor samples where loss in intron 1 occurred (n = 56), 96.4% of the matched normal tissue samples also demonstrated loss in intron 1.

These result shifted our focus to copy number loss in the ERBB4 gene only in GBM tumor samples. When compared to EGFR and PTEN loss in GBM tumor samples, which again were used as positive and negative controls, the 15.4% frequency of ERBB4 copy number loss behaves qualitatively similarly to that of GBM tumor suppressor PTEN (Fig 3a). Additionally, we analyzed copy number distributions of ERBB4 and housekeeping gene beta-tubulin (TUBB) from GBM samples in TCGA to demonstrate that, when compared to a gene centered at copy number of 2 with no loss or gain, ERBB4 exhibits notable copy number variation (S2 Fig).

Segmented copy number data available in TCGA allows us to localize copy number variations not only to whole genes but also to segments of nucleotides within genes. Comparing copy number to segments of nucleotides within the ERBB4 gene revealed that copy number loss in ERBB4 seems to be localized to one 5 kb region located in Intron 1 (Fig 3b). In fact, of the 81 GBM tumor samples where ERBB4 loss was observed, 69.1% of the loss was observed in this specific region. While this suggested that ERBB4 may demonstrate tumor suppressor activity that is compromised when a 5 Kb region in Intron 1 is deleted, we found that 96.4% of the matched normal tissue samples for these patients also demonstrated copy number loss in intron 1 (Fig 3c). Thus, this ERBB4 CNV is likely not a *de novo* somatic mutation that is a driver of GBM.

### Frequency of ERBB4 copy number loss in the general population

Although the clinical data do not support the initial suggestion from CCLE data that ERBB4 copy number loss is associated with GBM, it may be possible that loss in intron 1 of the ERBB4 gene is a factor that increases the risk that an individual will develop GBM. To investigate the possibility that this CNV may still be a predisposing factor to GBM, its frequency was characterized in the general, non-GBM population using consolidated population data from the DECIPHER database [25]. Querying “ERBB4” in DECIPHER’s genome browser returns common copy number variants observed within this gene in the general population in the *Population*: *Copy-Number Variants* track. Different studies used to obtain copy number information for the general population are merged into this database and separated by study. We used data from the *Affy6* study only (n = 5919), which was generated using the same Affymetrix SNP 6.0 microarray platform as was used in CCLE and TCGA as a part of the Sanger Institute’s Wellcome Trust Case Control Consortium (WTCCC) study [26].

**Fig 4:**
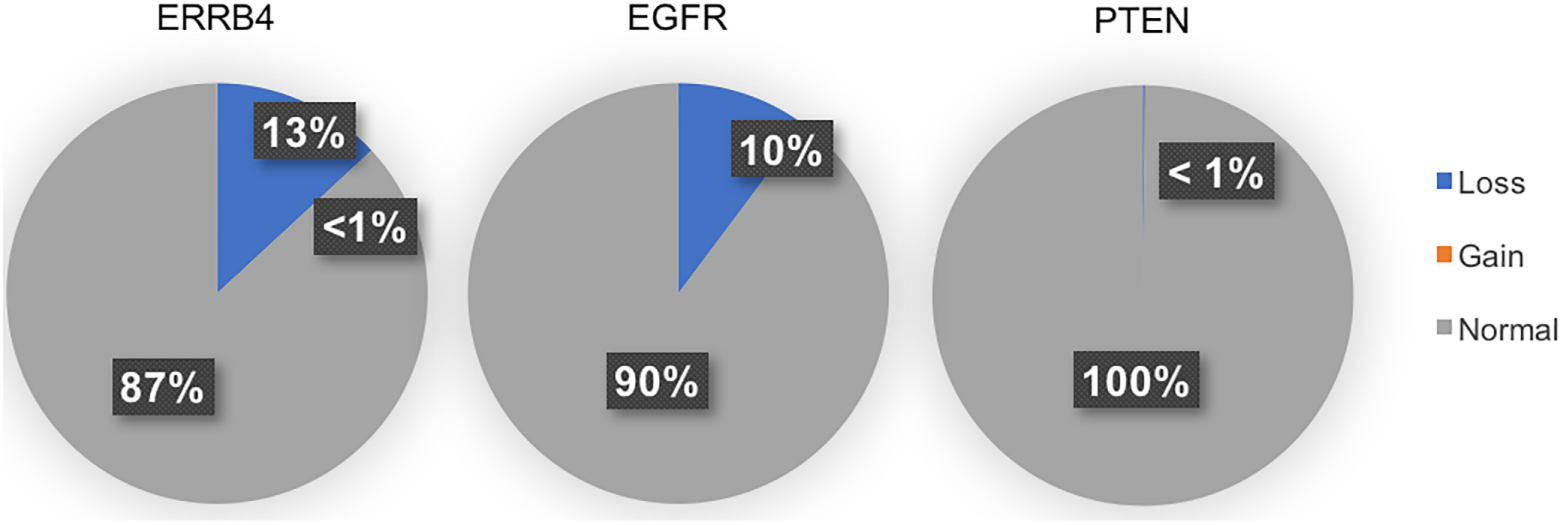
Frequency of copy number loss in the general population. Shown are the percentages of normal copy number, copy number loss, and copy number gain of the ERBB4 gene compared to known de novo driver mutations in GBM EGFR and PTEN in the general, healthy population from the DECIPHER database (n = 5919). Unlike in de novo driver mutations, the ERBB4 CNV we observed occurs at a similar frequency in the general population (12.5%) as it does in the GBM population (15.4%).

The frequency of CNV in intron 1 of the ERBB4 gene compared to instances of CNV in the EGFR and PTEN genes in the general population is depicted in Fig 4. It was found that the CNV occurs at a similar frequency in the general population (12.5%) as it does in the GBM population (15.4%). Comparison to the frequency of EGFR and PTEN CNV’s in the general, non-GBM population confirms that *de novo* driver mutations do not occur at the same frequency in the general population as they do in the disease population. *De novo* mutations demonstrate little CNV in the general population and increased CNV in the disease population. From this result, we concluded that loss in the ERBB4 gene is not a predisposing factor to GBM.

## Discussion

The primary goal of this manuscript is to caution that, while publicly available copy number data from databases such as CCLE may motivate interesting research questions, it is important to corroborate these findings by also looking at copy number data from patients in the disease population as well as the general healthy population as cell lines may contain artefactual findings. A deeper investigation of copy number data in tumor tissue samples from GBM patients in TCGA and the general, healthy population in DECIPHER disproved our initial hypothesis that ERBB4 may be acting as a tumor suppressor in GBM. We attribute this artefactual initial finding from glioma cell lines in CCLE to a number of possible factors including the known limitations associated with both cell line studies [27], the analysis of only gene-level and not segment-level data, as well as the lack of matched normal tissue data with which to compare CNV’s in cell lines. In addition, we noticed much variation in the resolution of base pair segments between patients and genes while analyzing copy number data generated using an aCGH microarray platform. For example, Patient 1 may have multiple ERBB4 copy number values because the ERBB4 gene spanned multiple segments in the microarray (i.e. higher resolution) while Patient 2 may have one copy number value that describes not only the ERBB4 gene but also other neighboring genes within the same chromosome because the microarray segment contained multiple genes (i.e. lower resolution). Use of more recently developed whole genome sequencing technology to infer copy number would most likely address copy number resolution issues mentioned here [28].

A secondary goal of this work is to offer a comprehensive methodology for using publicly available copy number data from CCLE, TCGA, and DECIPHER to infer the role of CNV’s in cancer progression. The usefulness of this technique in forming research questions related to studying cancer can be illustrated by looking at the copy number analysis results for the positive and negative controls used in this study. EGFR and PTEN, genes we know to act as an oncogene and tumor suppressor respectively in GBM, demonstrate expected behavior in our copy number analysis. That is, copy number gain is observed for EGFR and copy number loss is observed for PTEN. While ERBB4 CNV in glioma cell lines turned out to be an artifact, that is not to say that other CNV’s important in the development of cancer may not be uncovered using this method. As mentioned in the introduction, ERBB4 mutations have been linked to different cancer types. It would be interesting to use the methods stated here to analyze CNV patterns of ERBB4 in other types of cancer with which it has been associated e.g. melanoma, medulloblastoma, and breast cancers. This would further validate the relevance of using this technique in studying cancer.

## Supporting information

S1 Table CCLE and TCGA raw copy number data. Copy number by gene for 60 glioma cell lines downloaded from the Cancer Cell Line Encyclopedia (CCLE) data portal and copy number segmented data for 526 GBM tumor samples downloaded from The Cancer Genome Atlas (TCGA) data portal.
(XLSX)

S1 Fig Frequency of ERBB4 copy number loss in HGCC cell lines. Copy number data from the HGCC cited in *Xie et al*. *EBioMedicine*, *2015* showed normal copy number for ERBB4 across 48 GBM cell lines.
(TIFF)

S2 Fig Copy number frequency distributions of ERBB4 and housekeeping gene TUBB in TCGA. When compared to a housekeeping gene, beta-tubulin (TUBB), centered at 2 with no loss or gain, ERBB4 exhibits notable copy number variation. Copy number loss of ERBB4 occurs in 15.4% of samples, while copy number loss of beta-tubulin occurs in only 2.4% of samples. Thresholds defining copy number loss and gain are represented by two black vertical lines.
(TIFF)
