# Supplementary figures and images for: Analysis of copy number loss of the ErbB4 receptor tyrosine kinase in glioblastoma
Source: PLoS One. 2018 Jan 17;13(1):e0190664. doi: 10.1371/journal.pone.0190664 (PMC5771580; doi:10.1371/journal.pone.0190664)

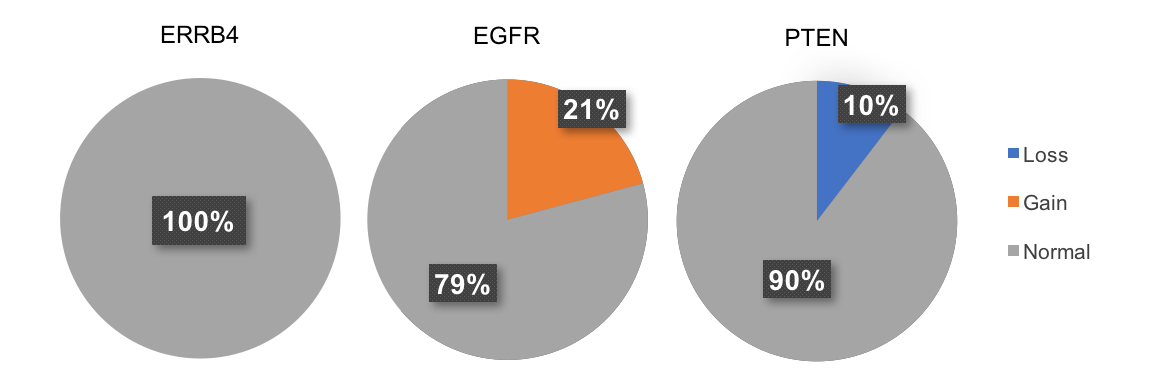

Supplement: S1 Fig — Copy number data from the HGCC cited in Xie et al. EBioMedicine, 2015 showed normal copy number for ERBB4 across 48 GBM cell lines. (TIFF) [file pone.0190664.s002.tiff]

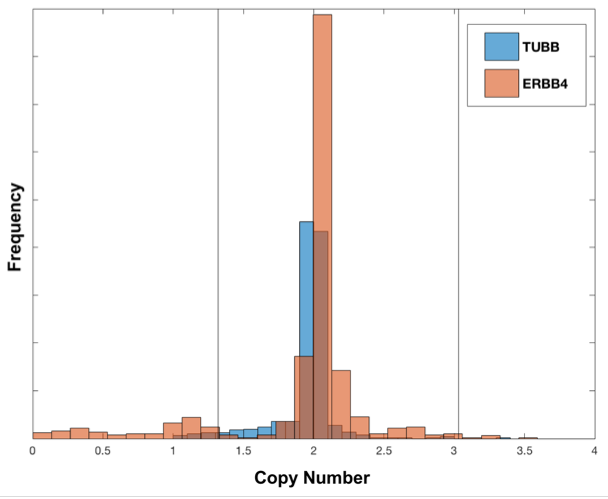

Supplement: S2 Fig — When compared to a housekeeping gene, beta-tubulin (TUBB), centered at 2 with no loss or gain, ERBB4 exhibits notable copy number variation. Copy number loss of ERBB4 occurs in 15.4% of samples, while copy number loss of beta-tubulin occurs in only 2.4% of samples. Thresholds defining copy number loss and gain are represented by two black vertical lines. (TIFF) [file pone.0190664.s003.tiff]
